# Secular trends in body image dissatisfaction and associated factors among adolescents (2007–2017/2018)

**DOI:** 10.1371/journal.pone.0280520

**Published:** 2023-01-19

**Authors:** Isadora Gonzaga, Marina Ribovski, Gaia Salvador Claumann, Alexandra Folle, Thais Silva Beltrame, Maria Fernanda Laus, Andreia Pelegrini

**Affiliations:** 1 Department of Physical Education, Santa Catarina State University, Florianópolis, Santa Catarina, Brazil; 2 Department of Nutrition, University of Ribeirão Preto, Ribeirão Preto, São Paulo, Brazil; Universidade Federal de Santa Catarina, BRAZIL

## Abstract

**Objective:**

To assess secular trends in body image dissatisfaction and associated factors among adolescents over a 10-year period (2007-2017/2018).

**Methods:**

The sample included 1,479 (2007, *n* = 531; 2017/2018, *n* = 948) high school adolescents of both sexes, aged 14 to 18 years, of schools in a city in southern Brazil, in 2007 and 2017/2018. Data were collected on sex, age, physical activity (IPAQ, short form), anthropometric measurements (body weight, height, and triceps and subscapular skinfolds), and body image dissatisfaction (figure rating scale). The variables were compared with Independent samples *t*-test and Mann–Whitney *U*-test. The associations between sex and other variables were examined with chi-square test, and the factors associated with body image were identified with multinomial logistic regression.

**Results:**

Most adolescents were dissatisfied with their body image in 2007 (65,2%) and 2017/2018 (71,1%). The prevalence of body dissatisfaction increased by 9.2% over the 10-year period, particularly dissatisfaction with thinness (21.3%). After stratification by sex, in 2017/2018 sample, dissatisfaction with overweight was 46.1% greater in boys, and dissatisfaction with thinness was 66.9% greater in girls. Factors found to be significantly associated with body image dissatisfaction were physical activity level, and body adiposity.

**Conclusions:**

There was a secular trend toward increased body image dissatisfaction in both sexes. Future efforts should go beyond scientific production, in public and private environments aimed at increasing awareness of health issues related to body care in the physical, psychological, and environmental domains.

## Introduction

Body image is a complex, multidimensional construct, and includes experiences related to physical appearance, such as perceptions and attitudes [1]. Historically, most studies on the topic have focused on identifying and understanding body dissatisfaction [2,3], which comprises the dimension of body appraisal (one of the attitudinal components of body image) [1], including Brazilian investigations [4–6]. The interest in investigating body dissatisfaction in the general and, in particular, in the adolescence population stems from the association of this component with several negative consequences for physical and mental health. Such outcomes include low self-esteem and disordered eating [7], common mental disorders such as depression and anxiety [8], and even suicide attempts [9].

Given these effects, the high prevalence of body dissatisfaction observed in Brazilian adolescents during the last decade is worrisome. In a study with Brazilian adolescents aged 12 to 17 years, 45% of the participants were found to be dissatisfied with their body weight; dissatisfaction was higher among girls (53.8%) than boys (36.4%) [8]. In southern Brazil, the prevalence of body dissatisfaction was 75.2% among adolescents, with 79.5% of girls and 70.3% of boys reporting dissatisfaction [6]. These prevalence rates are critical not only because of their high values but also because there seems to be a tendency for the number of Brazilian adolescents who are dissatisfied to remain or increase over the years.

In other countries, as the United States of America, Australia, Lithuania and Cyprus, there have been studies of secular trends on adolescent body image [10–14]. In the United States of America, there was a positive trend in body image dissatisfaction in males between 1999 and 2010 [10,11], while in females there was no difference [10], but when they were analyzed stratified by weight status, eutrophic females showed a negative trend in body image dissatisfaction [11]. In Cyprus, no difference in body image dissatisfaction was observed in male and female adolescents between 2003 and 2010 [14]. In studies with female adolescents, Australian students showed a positive trend in body image dissatisfaction [12], while in Lithuanian adolescents, this trend was negative [13].

To our knowledge, no study has yet investigated secular trends in body image dissatisfaction among Brazilian adolescents. Secular trend analysis is the assessment of variations (e.g., increase, decrease, or stability) in phenomena, diseases, incidence and prevalence rates, and other health-related behaviors over several years or decades [15]. This type of analysis is considered one of the oldest and most valuable methods and is of primary importance in public health research [15].

Despite the extensive literature on body dissatisfaction in adolescents, there is a lack of information about its temporal variation and possible associated factors in Brazil. Thus, studies analyzing related health behaviors are of great value for understanding, performing interventions, and promoting behavioral changes in target groups as well as serving as a basis for future approaches. This study aimed to assess secular trends in body image dissatisfaction and associated factors in a sample of Brazilian adolescents over a 10-year period (2007–2017/2018).

## Material and methods

This is an epidemiological, school-based, descriptive study with a repeated cross-sectional design (2007–2017/2018). Two surveys were conducted, the first between July to December 2007 and the second from August to December 2017 and from March to May 2018. The surveys were approved by the Human Research Ethics Committees of the Federal University of Santa Catarina (2007 survey, UFSC protocol No. 372/2006) and the Santa Catarina State University (2017/2018 survey, UDESC protocol No. 2.172.699).

### Study population and sample

The study population comprised high school adolescents aged 14 to 18 years enrolled in state schools in Florianópolis, Santa Catarina, Brazil. Schools were selected based on the five municipal regions (downtown, north, east, south, and mainland), defined by the Florianópolis Municipal Health Department. The total number of schools in each municipal region was determined, and the school with the largest number of students was selected in an intentionally non-probabilistic manner, due to the fact that larger schools are more likely to better represent the diversity of each region.

According to information provided by the Santa Catarina State Department of Education, there were 12,741 adolescents enrolled in public high schools in 2007 and 10,192 adolescents in 2017. For both surveys, the sample size was calculated for a finite population, with the equation $n=Z_{\frac{\alpha }{2}}^{2}N(1-P)÷\varepsilon_{r}^{2}P(N-1)+Z_{\frac{\alpha }{2}}^{2}(1-P)$, and the confidence level used was 1.96, with a tolerable error of four percentage points, and estimated prevalence of 50% to unrecognized outcome [16]. An additional 10% was considered to compensate for losses, totaling a minimum sample size of 631 individuals for the 2007 survey and 936 individuals for the 2017/2018 survey. Eligibility criteria were the same for both surveys: high school students of both sexes, aged 14 to 18 years, who were regularly enrolled in one of the selected schools, were present at school at the time of data collection, provided written informed consent to participate, handed in the informed consent form signed by one of the parents or legal guardians, and did not have any condition that could prevent participation in physical assessments.

A total of 2,172 adolescents participated in the study. Participants who did not answer questions regarding body image dissatisfaction (*n* = 304), or physical activity (*n* = 50), as well as those who were older than 18 years (*n* = 63) or did not complete or perform skinfold thicknesses measurements (*n* = 225) were excluded. Thus, the final sample comprised 1,479 adolescents: 531 participated in the first survey (2007) and 948 in the second survey (2017/2018). There were 821 girls and 658 boys.

### Study variables

Body image dissatisfaction, considered as a dependent variable in the current study, was measured using a figure rating scale [17] validated for the Brazilian population [18]. The scale consists of nine female and nine male silhouettes, ranging from extreme thinness to severe obesity. Students were asked to indicate which image best represents their current (actual) silhouette and which image reflects the body image they wished they had (ideal). Body satisfaction scores were calculated as ideal figure minus actual figure. Students were then classified as follows: satisfied (satisfaction score = 0), dissatisfied with thinness (satisfaction score < 0), and dissatisfied with overweight (satisfaction score > 0).

The independent variables analyzed included physical activity level, and body adiposity (skinfold thicknesses). Information on age (full years) and sex assigned at birth (female or male) was self-reported.

Data on adolescents’ physical activity levels during the previous 7 days were collected using the International Physical Activity Questionnaire (IPAQ, short form), validated in a sample of Brazilian adolescents [19]. Participants were considered active if they engaged in moderate and/or vigorous physical activity for at least 60 min daily [20], otherwise, they were classified as insufficiently active.

Body weight and height were measured according to standard procedures [21]. Height measurements were obtained by using a metal measuring tape attached to a wall in the 2007 survey and a portable stadiometer (Sanny, São Bernardo do Campo, Brazil) in the 2017/2018 survey. Body weight was measured using a Plenna digital scale in 2007 and a Tanita digital scale in 2017/2018.

Triceps and subscapular skinfold thicknesses were measured using a Cescorf scientific skinfold caliper in both surveys. Three non-consecutive measurements were performed at each point. Body fat was estimated by the sum of the mean values of skinfold thicknesses and used to classify adolescents as low/normal or high adiposity [22]. Given the small number of adolescents with low body adiposity, low and normal adiposity individuals were grouped for statistical purposes.

### Data collection

Data collection was authorized by the Regional Education Management Body (GERED) of Florianópolis, Santa Catarina State. Researchers presented the project at selected schools, and permission was obtained from school directors and/or coordinators. Classrooms were selected by drawing lots, and data were gathered on school premises. Adolescents answered the questionnaire in their classrooms, and anthropometric measurements were carried out in a separate room. All collections were performed during class periods with the permission of teachers.

### Statistical analysis

Descriptive (mean, standard deviation, and frequency distribution) and inferential analyses were performed using IBM SPSS Statistics version 20.0. The Kolmogorov–Smirnov test was used to examine the normality of data distribution. The Independent samples *t*-test and Mann–Whitney *U*-test were applied to compare the variables. The chi-square test was used to examine possible associations of sex with body image dissatisfaction, body adiposity, and physical activity. As the dependent variable (body image dissatisfaction) had three categories, multinomial logistic regression was used to identify associated factors; the reference category was body image satisfaction. Odds ratios (OR) and 95% confidence intervals (CI) were estimated in crude and adjusted analyses for independent variables (age, body adiposity, and physical activity level). The level of significance was set at 5%.

## Results

In 2007, significant sex differences were observed in all independent variables (body weight, height, triceps skinfold thickness, subscapular skinfold thickness, skinfold sum, and physical activity), except age. Girls had higher mean values on triceps skinfold thickness, subscapular skinfold thickness, and skinfold sum, whereas boys had higher mean values on body weight, height, and physical activity. In 2007 and 2017/18, sex was associated with all variables (body adiposity, physical activity, and body image dissatisfaction) (Table 1).

**Table 1:** General characteristics of high school adolescents included in the 2007 and 2017/2018 surveys of body image dissatisfaction. Florianópolis, Santa Catarina State, Brazil (2018).

Variable	2007				2017–2018			
	Total	Boys	Girls	*p*-value	Total	Boys	Girls	*p*-value
	$\bar{x}$ (SD)	$\bar{x}$ (SD)	$\bar{x}$ (SD)		$\bar{x}$ (SD)	$\bar{x}$ (SD)	$\bar{x}$ (SD)	
Age (years)	16.06 (0.99)	16.05 (1.06)	16.06 (0.96)	0.914	16.44 (0.98)	16.53 (1.01)	16.34 (0.95)	0.005
Body weight (kg)	57.75 (11.28)	63.65 (10.56)	54,98 (10.53)	<0.001	62.56 (12.95)	66,80 (12,81)	58.06 (11.51)	<0.001
Height (m)*	1.65 (0.09)	1.73 (0.07)	1.61 (0.06)	<0.001*	1.72 (0.09)	1.77 (0.07)	1.65 (0.06)	<0.001*
Triceps skinfold thickness (mm)	14.87 (6.45)	10.03 (5.03)	17.15 (5.75)	<0.001	14.17 (7.20)	10.66 (5.90)	17.90 (6.56)	<0.001
Subscapular skinfold thickness (mm)	13.27 (6.61)	10.45 (6.45)	14.60 (6.27)	<0.001	12.66 (6.94)	10.72 (6.58)	14.72 (6.72)	<0.001
Skinfold sum (mm)	28.15 (12.27)	20.48 (10.88)	31.75 (11.20)	<0.001	26.84 (13.31)	21.39 (11.60)	32.61 (12.55)	<0.001
Physical activity (min/day)	122.57 (170.06)	170.21 (235.49)	100.14 (122.35)	<0.001	95.26 (140.26)	126.45 (164.17)	62.16 (99.28)	<0.001
**Variable**	***n* (%)**	***n* (%)**	***n* (%)**	***p*-value**	***n* (%)**	***n* (%)**	***n* (%)**	***p*-value**
Body image dissatisfaction^+^				<0.001				<0.001
Satisfied	185 (34.8)	47 (25.4)	138 (74.6)		274 (28.9)	149 (54.4)	125 (45.6)	
Dissatisfied with thinness	135 (25.4)	88 (65.2)	47 (34.8)		292 (30.8)	192 (65.8)	100 (34.2)	
Dissatisfied with overweight	211 (39.7)	35 (16.6)	176 (83.4)		382 (40.3)	147 (38.5)	235 (61.5)	
Body adiposity^+^				0.001				0.003
Low/normal	398 (75.0)	143 (35.9)	255 (64.1)		695 (73.3)	378 (54.4)	317 (45.6)	
High	133 (25.0)	27 (20.3)	106 (79.7)		253 (26.7)	110 (43.5)	143 (56.5)	
Physical activity level^+^				0.017				<0.001
Active	288 (54.2)	105 (36.5)	183 (63.5)		423 (44.6)	275 (56.4)	148 (32.2)	
Insufficiently active	243 (45.8)	65 (26.7)	178 (73.3)		525 (55.4)	213 (43.6)	312 (67.8)	

Mann–Whitney *U*-test.

* Independent samples *t*-test. ^+^ Chi-square test. *p* < 0.05. $\bar{x}$, mean; SD, standard deviation.

**Fig 1:**
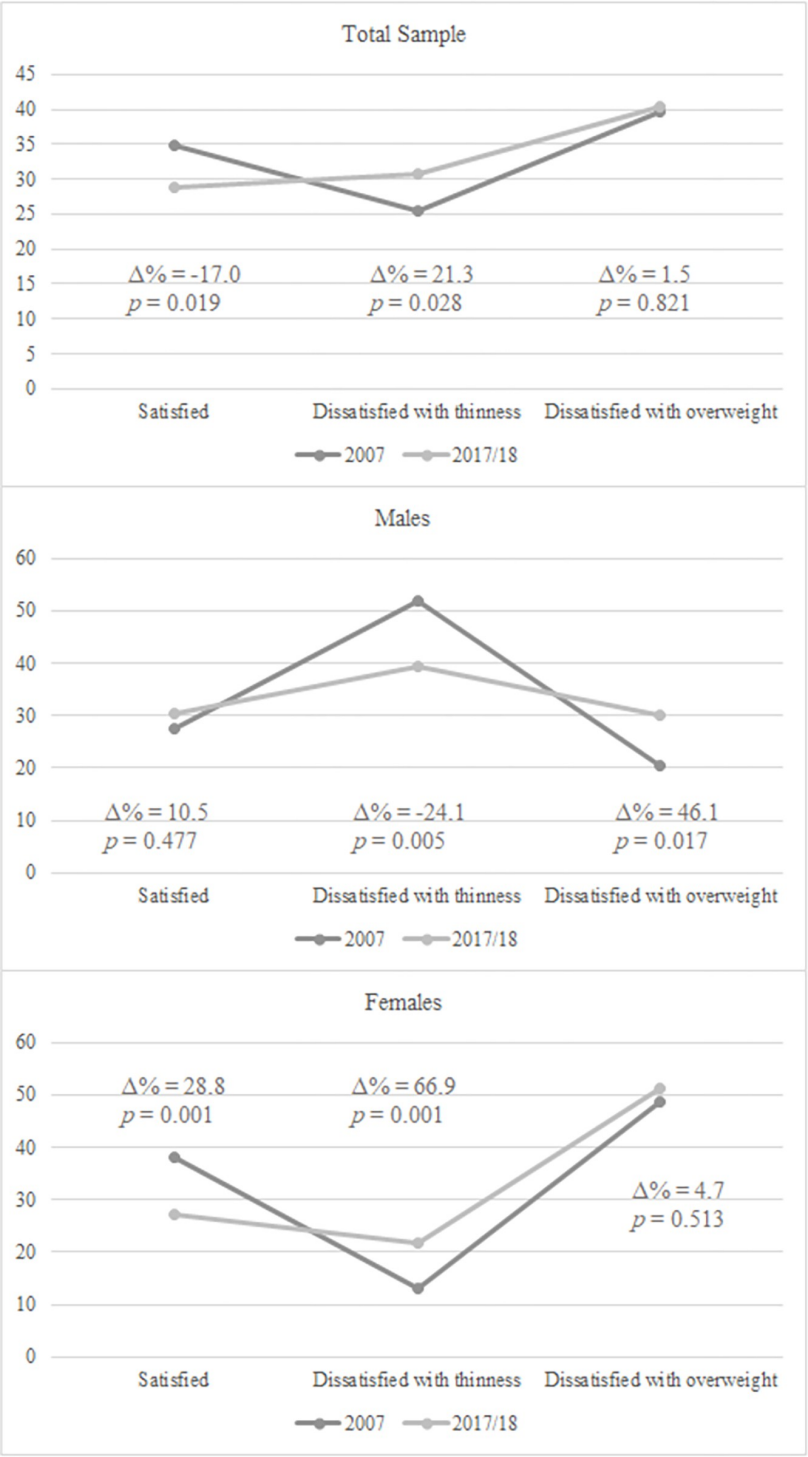
Prevalence of body image dissatisfaction among high school adolescents enrolled in public schools according to survey year (2007 and 2017/2018). Florianópolis, Santa Catarina State, Brazil (2018).

The prevalence of body image dissatisfaction in the total sample in 2007 was 65.1% (25.4% were dissatisfied with thinness and 39.7% with overweight). Whereas boys were more dissatisfied with thinness (51.8%), girls were more dissatisfied with overweight (48.8%). In 2017, body image dissatisfaction was reported in 71.1% of the sample (30.8% reported dissatisfaction with thinness and 40.3% with overweight). Most boys were dissatisfied with thinness (39.3%), and girls showed greater dissatisfaction with overweight (51.1%) (Fig 1).

In the total sample, from 2007 to 2017/2018, body image satisfaction was 17% smaller, and body image dissatisfaction was 9.2% greater. Dissatisfaction with thinness was 21.3% greater in 2017/2018, and no changes in dissatisfaction with overweight were observed over the decade (*p* = 0.821).

Among boys, from 2007 to 2017/2018, body image dissatisfaction with thinness was 24.1% (*p* = 0.005) smaller, and dissatisfaction with overweight was 46.1% (*p* = 0.017) greater. Among girls, body image dissatisfaction was 28.8% (*p* = 0.001) smaller in 2017/2018, dissatisfaction with thinness with overweight were 66.9% (*p* = 0.001) greater, while no changes in dissatisfaction with overweight were observed from 2007 to 2017/2018 (*p* = 0.513).

Both crude and adjusted analyses of 2007 survey data (Table 2) showed that only body adiposity was associated with dissatisfaction with overweight in boys. Adolescents with high body adiposity were 24.87 times (95% CI = 6.07–101.91) more likely to be dissatisfied with overweight than adolescents from low/normal adiposity. In the 2017/2018 survey, crude and adjusted analyses showed an association of body adiposity and physical activity with dissatisfaction with thinness. Boys with high body adiposity were less likely to be dissatisfied with thinness (OR = 0.16, 95% CI = 0.05–0.50), and boys with insufficient levels of physical activity were 66% (95% CI = 1.06–2.60) more likely to experience the same condition. On the other hand, adolescent boys with high body adiposity and those who had insufficient levels of physical activity were 13.56 (95% CI = 7.20–25.56) and 1.77 (95% CI = 1.03–3.05) times, respectively, more likely to be dissatisfied with overweight than boys with low/normal body adiposity and who were physically active.

**Table 2:** Associations between body image dissatisfaction and general characteristics of male adolescents enrolled in public high schools in Florianópolis, Santa Catarina, Brazil, in 2007 and 2017/2018.

Variable	2007				2017/2018			
	Dissatisfaction with thinness		Dissatisfaction with overweight		Dissatisfaction with thinness		Dissatisfaction with overweight	
	OR (95% CI)	OR (95% CI)^#^	OR (95% CI)	OR (95% CI)^#^	OR (95% CI)	OR (95% CI)^#^	OR (95% CI)	OR (95% CI)^#^
Body adiposity								
Low/normal	1	1	1	1	1	1	1	1
High	0,34 (0,06–2,12)	0,27 (0,04–1,74)	**24,82 (6,40–96,29)**	**24,87 (6,07–101,91)**	**0,18 (0,06–0,54)**	**0,16 (0,05–0,50)**	**13,13 (7,09–24,29)**	**13,56 (7,20–25,56)**
Level of physical activity								
Active	1	1	1	1	1	1	1	1
Insufficiently active	0,64 (0,31–1,32)	0,67 (0,31–1,44)	0,83 (0,34–2,01)	0,74 (0,24–2,26)	**1,61 (1,03–2,50)**	**1,66 (1,06–2,60)**	**2,18 (1,36–3,48)**	**1,77 (1,03–3,05)**

Multinomial regression. ^#^Adjusted for all independent variables and age. OR, odds ratio; CI, confidence interval.

In girls, in both surveys, there was an association of physical activity level with dissatisfaction with thinness and of body fat with dissatisfaction with overweight (Table 3). Insufficiently active girls (2007: OR = 2.32, 95%CI = 1.16–4.62; 2017/2018: OR = 2.34, 95%CI = 1.30–4.21) were more likely to be dissatisfied with thinness than active girls. Girls with high body adiposity (2007: OR = 11.41, 95%CI = 5.86–22.23; 2017/2018: OR = 12.17, 95%Ci = 6.28–23.59) were more likely to be dissatisfied with overweight. Also, girls with high body adiposity were less likely to be dissatisfied with thinness (2017/18: OR = 0,19; 95%CI = 0,04–0,88).

**Table 3:** Associations between body image dissatisfaction and general characteristics of female adolescents enrolled in public high schools in Florianópolis, Santa Catarina, Brazil, in 2007 and 2017/2018.

Variable	2007				2017/2018			
	Dissatisfaction with thinness		Dissatisfaction with overweight		Dissatisfaction with thinness		Dissatisfaction with overweight	
	OR (95% CI)	OR (95% CI)^#^	OR (95% CI)	OR (95% CI)^#^	OR (95% CI)	OR (95% CI)^#^	OR (95% CI)	OR (95% CI)^#^
Body adiposity								
Low/normal	1	1	1	1	1	1	1	1
High	0,47 (0,10–2,17)	0,47 (0,10–2,21)	**11,50 (5,93–22,29)**	**11,41 (5,86–22,23)**	**0,19 (0,04–0,88)**	**0,19 (0,04–0,88)**	**11,46 (5,99–21,91)**	**12,17 (6,28–23,59)**
Level of physical activity								
Active	1	1	1	1	1	1	1	1
Insufficiently active	**2,36 (1,19–4,68)**	**2,32 (1,16–4,62)**	1,37 (0,88–2,15)	1,28 (0,77–2,14)	**2,26 (1,26–4,03)**	**2,34 (1,30–4,21)**	1,61 (1,03–2,53)	1,35 (0,81–2,24)

Multinomial regression.

^#^Adjusted for all independent variables and age. OR, odds ratio; CI, confidence interval.

## Discussion

This study aimed to assess secular trends in body image dissatisfaction and associated factors among adolescents from 2007 to 2017/2018. In both surveys, most adolescents reported being dissatisfied with their body image. In 2007, the prevalence of body image dissatisfaction was 65.1%, whereas, in 2017, the prevalence was 71.1%. Over the study decade, dissatisfaction was 9.2% greater, and satisfaction with body image was 17% smaller.

Several studies conducted during this period showed similar results regarding the prevalence of body dissatisfaction [8], including in southern Brazil [23,24]. These results reinforce the tendency of adolescents to become increasingly dissatisfied with their bodies. A plausible explanation is that adolescence is characterized by successive body modifications; this phase of life is one of the most susceptible to body dissatisfaction due to adolescents’ strong focus on physical appearance [25]. In addition, adolescents are more likely to compare themselves and their peers to other individuals, which may influence their perception of physical appearance, representing a decisive factor in the development of body image [26].

Another possible explanation for the increase in body dissatisfaction over a 10-years period may be related to the decreased level of physical activity, which is intrinsically linked to the increase in screen time among adolescents in recent years [27]. Consumption of social media while using the Internet becomes common [28], and adolescents who use social media more frequently tend to report higher levels of body dissatisfaction [29]. The use of social media leaves adolescents exposed to appearance-related messages, causing appearance ideals to be disseminated to be internalized and also result in comparisons, which consequently results in body dissatisfaction [29].

The current study found that prevalence of dissatisfaction with thinness among adolescents was 21.3% higher in the 2017/2018 sample. This result may be related to the sociocultural context in which adolescents are embedded [30] and to the muscular body ideal imposed on boys [30] and girls [28]. Among boys, there was a negative trend of dissatisfaction with thinness (24.1%) over the 10 years. This finding may be explained by the tendency of boys to be more concerned with muscularity than thinness and, consequently, to exhibit behaviors that reflect a desire to achieve a more muscular physique [30]. In contrast, dissatisfaction with being overweight was 46.1% higher in 2017/2018 survey. These results may be linked to the overall increase in overweight/obesity in adolescents [31]. Study participants of both sexes showed higher body adiposity in 2017/2018 (Table 1), however the data were not significant. In part, these could be associated with increased in dissatisfaction with being overweight.

Among girls, the prevalence in body image satisfaction decreased by 28.2% over the 10-years period. In general, girls experience more social pressures related to appearance and body image than boys, reflecting on the higher body dissatisfaction experienced by girls [7]. Dissatisfaction with thinness was 66.9% higher in 2017/2018, while dissatisfaction with overweight did not vary significantly (4.7%). Several studies have observed an internalization of the thinness ideal in girls and women [32]; however, more recent studies reported changes in the body ideal towards a not-so-thin and more muscular body image [33].

Recent observations have shown that female adolescents show high dissatisfaction with their muscles [34,35]. Research conducted in Sweden revealed a 66% prevalence of muscle dissatisfaction among girls [34]. Although concern about body shape and weight is even more prevalent, concern about muscularity has been recurrent in girls in Germany [35]. Therefore, increased dissatisfaction with thinness among girls is likely associated with these trends, and it is important to note that in both surveys, the majority of girls dissatisfied with thinness are with normal body adiposity (95,7% and 98,0% respectively–**S3 Table**).

In line with studies investigated factors influencing body image in adolescents, the current study sought to identify factors associated with body image dissatisfaction in terms of both thinness and overweight. High body adiposity and low levels of physical activity were found to influence dissatisfaction. High body adiposity was related to dissatisfaction with overweight in both sexes in 2017/2018, corroborating data from the literature [26]. A study conducted with adolescents attending a public school in São José, Santa Catarina State, Brazil, reported similar results [6]. It is therefore believed that although boys and girls were dissatisfied with their body image, their perception of body image was close to their actual physical condition; our results (**S1 Table**) showed that most of the boys in 2007 with body image dissatisfaction due to overweight, have high body adiposity (62,9%), and this finding is the same for girls in 2007 (52,3%) and 2017 (54,9%—**S3 Table**). Similar results were obtained in studies comparing measured and perceived body mass index [26] as well as measured body mass index and self-perception of body weight in adolescents [8].

Dissatisfaction with overweight may also be explained by the drive for muscularity. Body weight status may affect boys’ capacity to achieve the athletic and muscular ideal, either by a lack of muscle mass or by excess body fat covering the muscles [35]. In girls, it is suspected that an increase in body fat during adolescence can lead to the coverage of muscles and reduction in muscle tone, stimulating the drive for muscularity [35] and consequently body dissatisfaction.

Girls (in both surveys) and boys (in the 2017/2018 survey) with insufficient levels of physical activity were more likely to experience dissatisfaction with thinness. In the second survey, insufficiently active boys were observed to have greater dissatisfaction with being overweight. These results may be explained by the fact that 53.6% of boys with high body adiposity did not meet physical activity recommendations (**S2 Table**). These results may be related to the above-mentioned factors and the fact that adolescents who do not have adequate levels of physical activity will not benefit from the resulting physical changes. High body dissatisfaction has been associated with low levels of physical activity [7,8]. Physical activity is highly important for controlling body adiposity [7] and muscle mass, two factors that may influence dissatisfaction with thinness in boys [30] and girls, possibly stimulated by recent body ideals shared on social media [32].

It is worth noting that dissatisfaction with thinness might be related to the drive for muscularity, especially in boys. A study conducted with adolescents in Germany revealed that boys with low and medium weight status had higher muscularity concern than boys with high weight status [35]. Skinny boys may become dissatisfied because of their lack of muscle mass and feelings of lower physical development [35]. This hypothesis was supported by the results of a study with Swedish adolescents, in which 79.3% of boys showed muscle dissatisfaction [34].

Body image is an important dimension in the psychological health of adolescents [26]; body image dissatisfaction in young populations can lead to the adoption of poor eating habits, such as high consumption of sweets and sugary drinks, low consumption of fruits and vegetables [36], skipping breakfast, low number of daily meals (in the case of dissatisfaction with overweight), and high consumption of dairy products, fats, and oils (in the case of dissatisfaction with thinness) [7,36]. Dissatisfied adolescents tend to have chronic diseases, psychological and somatic complaints, and few family meals and are more likely to be bullied, not accepted by peers at school, and not able to talk about problems with family and friends [36]. Therefore, given the many negative consequences that body dissatisfaction can have on the lives of adolescents, in the current phase and adulthood, this phenomenon becomes a relevant public health problem and should be considered when evaluating adolescent health.

We also observed a negative association between high body adiposity and dissatisfaction with thinness in girls in 2017/18. The result can be explained by the body changes that occur in adolescent girls, which result in a greater amount of body fat [35], as well as the internalization of the thin ideal, which occurs more frequently in girls [32], so it is clear that girls with high body adiposity could be dissatisfied with thinness. It’s important to point that in both surveys, most of girls dissatisfied with overweight have high body adiposity (52,3% and 54,9% respectively—supplementary data).

It is noteworthy that, to the best of our knowledge, this is the first study to investigate secular trends of body image dissatisfaction among adolescent students in Brazil. Our results may support the development of health education actions in the school environment aimed at reducing the prevalence of body dissatisfaction and mitigating the consequences of this phenomenon. A possible limitation of this study was the use of a self-administered questionnaire, which opens the possibility for misunderstanding and misinterpretation by participants. However, this instrument is widely applied in epidemiological research because of its ease of application and low cost. Although physical activity was self-reported, this method has been validated in adolescents and used in previous studies [37]. The figure rating scale has some limitations regarding the assessment of body image construct; nevertheless, it can be used for comparison purposes, as was performed here between surveys. The scale showed good validity and reproducibility for use in the Brazilian population [18]. Another limitation is that the anthropometric measurement tools used here were of different brands, but all instruments showed good precision.

## Conclusion

The 10-year secular trend analysis revealed an increase in prevalence of body image dissatisfaction among adolescents, with girls showing a positive trend toward dissatisfaction with thinness and boys having a positive trend toward dissatisfaction with overweight. Furthermore, body adiposity and physical activity (in both surveys) were significantly associated with the outcome variable.

## Supporting information

S1 Table Associations between body image dissatisfaction and body adiposity of male adolescents enrolled in public high schools in Florianópolis, Santa Catarina, Brazil, in 2007 and 2017/2018. (DOCX)

S2 Table Associations between body adiposity and physical activity of male adolescents enrolled in public high schools in Florianópolis, Santa Catarina, Brazil, in 2007 and 2017/2018. (DOCX)

S3 Table Associations between body image dissatisfaction and body adiposity of female adolescents enrolled in public high schools in Florianópolis, Santa Catarina, Brazil, in 2007 and 2017/2018. (DOCX)

S4 Table Associations between body adiposity and physical activity of female adolescents enrolled in public high schools in Florianópolis, Santa Catarina, Brazil, in 2007 and 2017/2018. (DOCX)
