# Supplementary material for: Secular trends in body image dissatisfaction and associated factors among adolescents (2007–2017/2018)
Source: PLoS One. 2023 Jan 19;18(1):e0280520. doi: 10.1371/journal.pone.0280520 (PMC9851498; doi:10.1371/journal.pone.0280520)
Supplement: S1 Table — (DOCX) [file pone.0280520.s001.docx]

**S1 Table.** Associations between body image dissatisfaction and body adiposity of male adolescents enrolled in public high schools in Florianópolis, Santa Catarina, Brazil, in 2007 and 2017/2018.

| Variables | **Body image dissatisfaction** | | | *p*-value |
| --- | --- | --- | --- | --- |
|  | **2007 n (%)** | | |  |
|  | Satisfied | Dissatisfied with thinness | Dissatisfied with overweight |  |
| **Body Adiposity** |  |  |  | <0,001 |
| Low/normal | 44(93.6) | 86(97.7) | 13(37.1) |  |
| High | 3(6.4) | 2(2.3) | 22(62.9) |  |
|  | **2017/2018 n (%)** | | |  |
|  | Satisfied | Dissatisfied with thinness | Dissatisfied with overweight |  |
| **Body Adiposity** |  |  |  | <0,001 |
| Low/normal | 133(89.3) | 188(97.9) | 57(38.8) |  |
| High | 16(10.7) | 4(2.1) | 90(61.2) |  |

Chi-square test.
