# Supplementary material for: Secular trends in body image dissatisfaction and associated factors among adolescents (2007–2017/2018)
Source: PLoS One. 2023 Jan 19;18(1):e0280520. doi: 10.1371/journal.pone.0280520 (PMC9851498; doi:10.1371/journal.pone.0280520)
Supplement: S2 Table — (DOCX) [file pone.0280520.s002.docx]

**S2 Table.** Associations between body adiposity and physical activity of male adolescents enrolled in public high schools in Florianópolis, Santa Catarina, Brazil, in 2007 and 2017/2018.

|  | 2007 n (%) | | *p*-value | 2017/2018 n (%) | | *p*-value |
| --- | --- | --- | --- | --- | --- | --- |
| Variables | **Body Adiposity** | |  | **Body Adiposity** | |  |
|  | Low/normal | High |  | Low/normal | High |  |
| **Physical Activity** |  |  | 0,465 |  |  | 0,011 |
| Active | 89 (62.2) | 16 (59.3) |  | 224 (59.3) | 51 (46.4) |  |
| Insufficiently active | 54 (37.8) | 11 (40.7) |  | 154 (40.7) | 59 (53.6) |  |

Chi-square test.
