# Supplementary material for: Secular trends in body image dissatisfaction and associated factors among adolescents (2007–2017/2018)
Source: PLoS One. 2023 Jan 19;18(1):e0280520. doi: 10.1371/journal.pone.0280520 (PMC9851498; doi:10.1371/journal.pone.0280520)
Supplement: S3 Table — (DOCX) [file pone.0280520.s003.docx]

**S3 Table.** Associations between body image dissatisfaction and body adiposity of female adolescents enrolled in public high schools in Florianópolis, Santa Catarina, Brazil, in 2007 and 2017/2018.

| Variables | **Body image dissatisfaction** | | | *p*-value |
| --- | --- | --- | --- | --- |
|  | **2007 n (%)** | | |  |
|  | Satisfied | Dissatisfied with thinness | Dissatisfied with overweight |  |
| **Body Adiposity** |  |  |  | <0,001 |
| Low/normal | 126(91.3) | 45(95.7) | 84(47.7) |  |
| High | 12(8.7) | 2(4.3) | 92(52.3) |  |
|  | **2017/2018 n (%)** | | |  |
|  | Satisfied | Dissatisfied with thinness | Dissatisfied with overweight |  |
| **Body Adiposity** |  |  |  | <0,001 |
| Low/normal | 113(90.4) | 98(98.0) | 106(45.1) |  |
| High | 12(9.6) | 2(2.0) | 129(54.9) |  |

Chi-square test.
